# Totally mechanical linear stapled anastomosis for minimally invasive Ivor Lewis esophagectomy: Operative technique and short‐term outcomes

**DOI:** 10.1111/1759-7714.13339

**Published:** 2020-02-03

**Authors:** Hui‐Jiang Gao, Ju‐Wei Mu, Wei‐Min Pan, Malcolm Brock, Mao‐Long Wang, Bin Han, Kai Ma

**Affiliations:** ^1^ Department of Thoracic Surgery The Affiliated Hospital of Qingdao University Qingdao China; ^2^ Department of Thoracic Surgery, National Cancer Center/National Clinical Research Center for Cancer/Cancer Hospital & Shenzhen Hospital Chinese Academy of Medical Sciences and Peking Union Medical College Shenzhen China; ^3^ Department of Anesthesia the Affiliated Hospital of Qingdao University Qingdao China; ^4^ Department of Surgery Johns Hopkins University Baltimore Maryland USA

**Keywords:** Anastomosis, complication, esophageal carcinoma, MIE

## Abstract

**Background:**

Anastomosis is one of the important factors affecting anastomotic complications after esophagectomy, and multiple reports have compared anastomotic complications among various techniques. However, there is insufficient evidence in the literature to definitively recommend one anastomotic technique over another.

**Method:**

We retrospectively evaluated 34 consecutive patients who underwent an improved totally mechanical side‐to‐side: posterior‐to‐posterior linear stapled (TM‐STS) technique for minimally invasive Ivor Lewis esophagogastric anastomosis, performed by a single surgeon between February 2015 to November 2017. The operative techniques and short‐term outcomes are analyzed in this study.

**Results:**

There were no conversions to an open approach and a complete resection was achieved in all patients undergoing this improved procedure. During the first half of the series, the median operation time was 355 minutes, ranging from 257 to 480 minutes. Over the second half of this series, the median operation time was reduced to 256 minutes. There were no mortalities or serious postoperative complications. Only one patient (2.9%) had an anastomotic leak, which resolved without intervention. Another patient (2.9%) experienced transient, delayed conduit emptying which upper gastrointestinal radiography determined was due to a mechanical obstruction caused by an abnormally long gastric tube in the chest cavity.

**Conclusions:**

The results of our study suggest that this improved TM‐STS technique is safe and effective for minimally invasive Ivor Lewis esophagectomy, and can be considered as one of the alternative procedure for patients with lower esophageal as well as Siewert types I/II gastroesophageal junction carcinoma.

## Introduction

For over a century, esophagectomy has been the mainstay of curative treatment for esophageal cancer. A significant number of patients have experienced long‐term survival following an esophagectomy for early‐stage esophageal cancer with low operative mortality.^1^ Due to the widespread application of neoadjuvant chemoradiotherapy as well as improvement of thoracoscopic surgical devices and techniques, minimally invasive esophagectomy (MIE) is increasingly being used to resect esophageal cancers.^2^ The development of minimally invasive esophageal surgery has accelerated and improved the rehabilitation of patients, reduced postoperative complications and mortality, and resulted in long‐term survivors.^3^ However, analogous to the open technique, anastomotic leakage and stricture formation following MIE procedures has been the most common and feared complication faced due to its potential for increasing operative mortality.^4^

As experience with MIE accumulates, there is a need to improve the MIE anastomotic technique. Methods of constructing the esophagogastric anastomosis have varied with the most common procedures advocated being hand sewn, circular stapled, linear stapled and modified Collard approach (combined linear and transverse stapled anastomosis). Different esophagogastric anastomotic techniques have their own characteristics, and multiple reports have compared anastomotic complications among various techniques.^5^, ^6^ A side‐to‐side anastomosis has been generally defined as a semi‐mechanical liner stapler procedure, and in several studies it has been shown to reduce the incidence of postoperative anastomotic strictures without increasing the rate of anastomotic fistulae.^6^, ^7^, ^8^, ^9^, ^10^ At present, there are relatively few studies using a mechanical anastomosis, especially as it is applied to cervical anastomoses during MIE procedures.^11^, ^12^ Importantly, there is insufficient evidence in the literature to definitively recommend one anastomotic technique over another.

Carcinoma of the distal esophagus and esophagogastric junction has been increasing in recently years.^13^ As a result, Ivor Lewis esophagectomy has been widely applied for patients with distal esophagus and esophagogastric junction carcinomas. However, the MIE Ivor Lewis esophagectomy is not frequently utilized compared with the open procedure, owing to the limitation of creating a safe, technically simple video‐assisted intrathoracic esophagogastric anastomosis. Because an anastomosis can be completed more reliably in the neck, most esophageal surgeons prefer the McKeown procedure instead of the Ivor Lewis esophagectomy in an MIE operation. Unfortunately, regardless of the technique employed, the incidence of anastomotic leaks is higher in the neck compared with those in the chest, as recently confirmed.^14^, ^15^

To overcome this issue, we have developed an improved “totally mechanical side‐to‐side: posterior‐to‐posterior linear stapled” (TM‐STS) technique for minimally invasive Ivor Lewis (MIE‐IL) esophagogastric anastomosis, designed to offer not only a wider anastomotic diameter and fewer morbidities associated with anastomotic leaks and strictures, but also technical simplicity in constructing an intrathoracic esophagogastric anastomosis. In this study, we describe an improved esophagogastric anastomotic technique in 34 MIE‐IL procedures, and review the postoperative outcome.

## Methods

This retrospective study was approved by the ethics committee of The Affiliated Hospital of Qingdao University. Informed consent was signed by all 34 patients or their legal representatives regarding the scientific use of collected data, including data from their medical records, obtained while undergoing a MIE‐IL esophagectomy using an TM‐STS esophagogastric anastomosis from February 2015 to November 2017. Demographic characteristics, perioperative outcomes and postoperative morbidity of the patients were also recorded. Since the data from this study did not include any patient identifying information, Institutional Review Board approval was not required.

All patients had a standardized, preoperative evaluation as previously described by Ben‐David *et al*.^14^ The enrolled patients had a resectable lower esophageal malignancy, Siewert type 1 or type 2 esophagogastric junction carcinoma. Those patients with a Siewert type 3 esophagogastric junction carcinoma were typically treated with a total or proximal gastrectomy, and excluded from this analysis. Preoperative tumor staging and nodal disease status were determined by dynamic enhanced computed tomography/positron emission tomography (CT/PET) scan or endoscopic ultrasound.^16^, ^17^ Minimally invasive Ivor Lewis esophagectomy using a gastric tube was offered as the operation of choice to all patients, except for those who had known requirements for a colonic conduit due to extensive previous abdominal or thoracic surgery.

### Surgical technique

**Figure 1:**
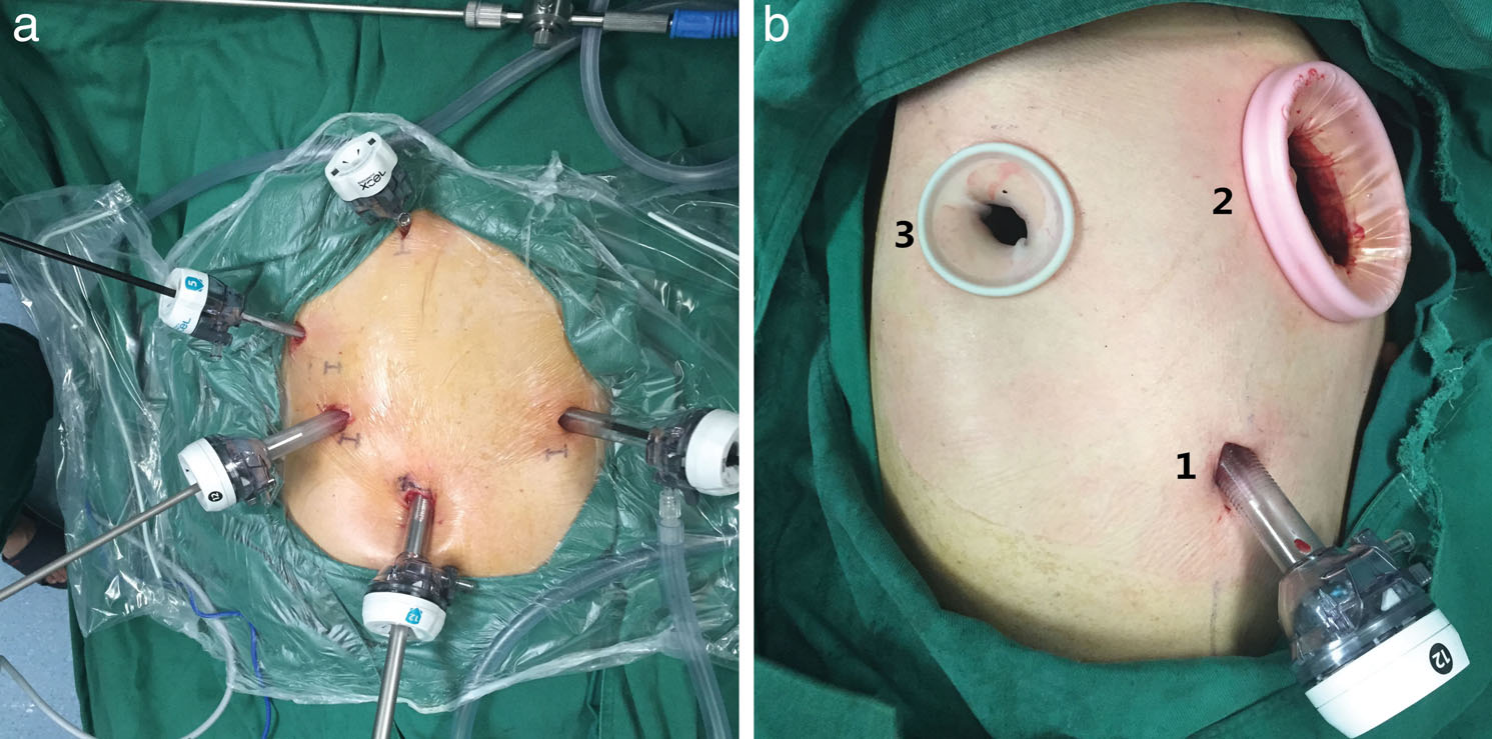
Operative photograph showing the port placements for the laparoscopic and thoracoscopic procedures. (**a**) Two 12 mm ports were placed in the left hypochondrium and in the right upper quadrant and two 5 mm ports in the left upper quadrant and below the xiphoid. A 12 mm camera port was placed just below the umbilicus. (**b**) One 12 mm port was placed in the seventh intercostal space in the right posterior axillary line and one 4 cm incision in the fourth intercostal space between the middle and posterior axillary line as the utility port. The 2 cm auxiliary port was placed in the seventh intercostal space in the right scapular line.

MIE‐IL is usually performed in two stages. First, laparoscopic stomach mobilization and intra‐abdominal lymphadenectomies are performed under general anesthesia, as previously described (Fig 1a).^18^, ^19^, ^20^ During this intra‐abdominal component of the mobilization, the procurement of the gastric conduit is initiated, but not completed with a bridge left at the fundus of the stomach. The jejunal nutrition tube is placed 30 cm distal to the Ligament of Treitz and a 3‐0 absorbable suture used to secure it to the abdominal wall with an intestinal canal.

Second, the patient is placed in the left lateral prone position. An esophagectomy is performed thoracoscopically using three access ports with the camera placed in the seventh intercostal space in the right posterior axillary line, and the utility port in the fourth intercostal space between the middle and posterior axillary line. The auxiliary port is placed in the seventh intercostal space in the right scapular line (Fig 1(b)B). The thoracoscopic procedure involves a mediastinal lymphadenectomy undertaken by removing lymph nodes under the carina, along the right and left recurrent laryngeal nerves, as well as adjacent to the main bronchus, the entire aortic arch and descending aorta, pulmonary ligament and diaphragm as well as adjacent to the paraesophageal lymph nodes. After the completion of lymphadenectomy and mobilization of the proximal esophagus, the gastric conduit is pulled up into the chest cavity through the hiatus with the staple line facing towards the surgeon as a landmark to prevent rotation of the conduit. The remaining bridge between the conduit and the specimen is then divided, following which the esophagus is transected above the level of the azygos arch with an Ethicon Stapler using a 60 mm endoscopic stapler (Ethicon Endo‐Surgery, Echelon Flex 60, “golden” tristapler cartridge).

**Figure 2:**
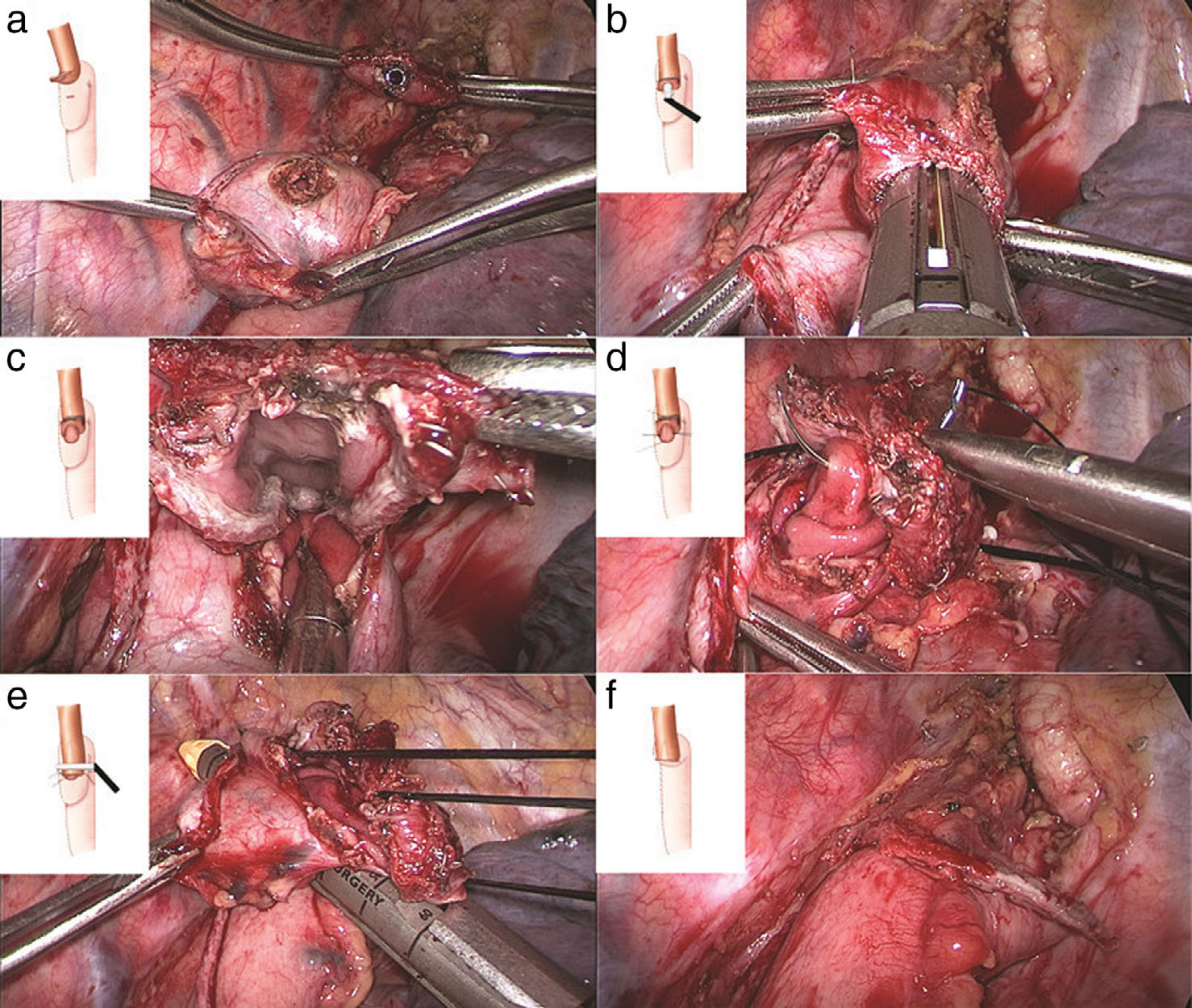
(**a**) The midposterior aspect of the upper third of the intrathoracic esophagus was aligned along the posterior gastric wall at the tip of the conduit with three initial tacking sutures placed approximately 2–3 cm apart. Two 5 mm rents were made in the stomach conduit and in the esophagus 1 cm away from the tacking sutures. (**b**) A 60 mm endoscopic stapler was fired cutting through the posterior wall of the gastric conduit and the redundant esophagus. Typically, only two‐thirds of the stapler length is used. (**c**) The inner view of the posterior wall of the side‐to‐side anastomosis. (**d**) The open common lumen was manually tacked with three sutures (two lateral sutures and one in the middle of the lumen) to create a line of traction. The lumen was closed with two layers of sutures beginning with inverted interrupted absorbable sutures. (**e**) The stapler was fired beneath the existing traction line to establish that the anterior wall of the gastric conduit and the redundant esophagus were aligned with a stay suture. (**f**) The LS side‐to‐side anastomosis was completed using two 60 mm golden cartridges (Ethicon Endo‐Surgery).

An intrathoracic totally mechanical side‐to‐side: posterior‐to‐posterior linear and transverse stapled (TM‐STS) anastomosis is then performed after removing the specimen from the utility port. The gastric conduit is brought up carefully until a sufficient length can be obtained for an esophagogastrostomy with the staple line facing towards the right side of the chest wall as a landmark for preventing the rotation of the conduit. Small incisions are made on the distal posterior wall of the esophageal stump and on the proximal posterior side of the gastric conduit 5 cm from the stump to make the TM‐STS anastomosis. A small incision on the conduit must be made transversely to prevent increasing the size of the anastomosis after closure of the common incision for insertion of the stapler. Three initial tacking sutures are placed between the adjacent conduit and esophageal walls beginning at the level of the transverse anastomosis. The anterior wall of the esophageal remnant and the remaining extroverted stump of the gastric conduit are then transected with a 60 mm golden cartridge (Ethicon Endo‐Surgery) 0.5 to 1 cm beneath the level of the tacking sutures. This is also performed through the fourth intercostal space port. Ultimately, a V‐shaped anastomosis is created by one or two 60 mm golden cartridges (Ethicon Endo‐Surgery) (Fig 2). After the thoracoscopic procedures are completed, a single 22‐French chest tube is inserted posteriorly next to, but not abutting, the esophagogastric anastomosis before chest closure.

**Figure 3:**
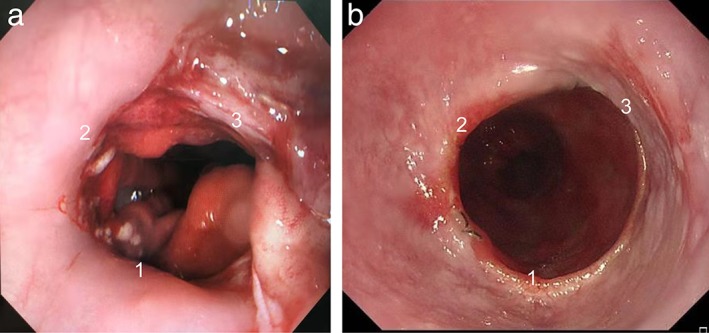
(**a**) Gastroscopic views of the anastomosis with three edges (1, 2, and 3) during the operation. (**b**) Gastroscopic views of the anastomosis seven months after surgery.

The reconstructed conduit is placed in the inherent space of the thoracic esophagus to reduce anastomotic tension. The anastomosis is then inspected using endoscopy to ensure patency and determine that no leak is present during insufflation of intraluminal air on submerging the anastomosis under fluid (Fig 3).

### Statistical analysis

Continuous variables were reported as the mean ranges and percentages were used for discrete characteristics. The improved TM‐STS technique for MIE‐IL anastomosis was the indicated treatment. All statistical analyses were performed using IBM SPSS Statistics for Windows, version 25.0 (IBM Corporation, Armonk, NY, USA).

## Results

Demographic characteristics for the 34 patients who underwent TM‐STS intrathoracic esophagogastric anastomosis are shown in Table 1. In summary, most patients (91.2%) were male with a median age of 62 years (range, 49–75 years). The median body mass index (BMI) was 22.4 kg/m^2^. A history of smoking and drinking were noted in 76.5% and 70.6% of patients, respectively. Although cessation of smoking was strongly recommended, current smoking was not a contraindication to surgery.

**Table 1:** Demographic characteristics of the entire cohort

Patient demographics	Value
Age (year), median (range)	62 (49–75)
Sex, *n* (%)	
Male	31 (91.2)
Female	3 (8.8)
BMI (kg/m^2^), median (range)	22.4 (16.4–35.7)
Smoking history, *n* (%)	
Yes	26 (76.5)
No	8 (23.5)
Alcohol use history, *n* (%)	
Yes	24 (70.6)
No	10 (29.4)
Comorbidities, *n* (%)	
Arrhythmia	10 (29.4)
Hypertension	4 (11.8)
Diabetes mellitus	0 (0)
Pulmonary function, median (range)	
FEV_1_%	108 (67–146)
DLCO%	96 (60–130)

BMI, body mass index; DLCO, carbon monoxide diffusing capacity; FEV1, forced expiratory volume in one second.

The perioperative data and pathological results are presented in Tables 2 and 3. There was no conversion to an open approach and complete resection was achieved in all patients undergoing this improved procedure. No serious complications resulting in an operative mortality occurred after any surgery using the new anastomotic technique. A total of 10 patients (29.4%) had a postoperative morbidity with three being secondary to respiratory complications. Only one of the 10 patients had a complication related to the improved anastomotic technique. During the first half of the series, the median operative time was 355 minutes (range 257 to 480 minutes). Over the second half of this series, the median operative time was reduced to 256 minutes. Postoperative complications included two patients with recurrent laryngeal nerve palsy with three of these patients developing aspiration pneumonia. Three further patients experienced atrial fibrillation, without heart failure and renal insufficiency. Other complications included one case of chylothorax and one case of delayed conduit emptying. With respect to anastomotic complications, only one patient (2.9%) had an anastomotic leak which resolved without intervention. Every patient had a jejunal feeding tube placed at the time of surgery and received intestinal nutrition two days postoperatively. The feeding tube was also used as a nutritional transition measure for oral intake after discharge especially for those who received adjuvant chemoradiotherapy. Finally, only one patient (2.9%) experienced a transient delayed conduit emptying due to a mechanical obstruction caused by an acute angle that developed in an abnormally long gastric conduit as it lay in the chest cavity, and as confirmed by upper gastrointestinal radiography.

**Table 2:** Operative characteristics of the entire cohort

Operative data	Value
Duration of surgery (minutes), median (range)	324 (185–480)
Perioperative blood loss (mL), median (range)	157 (50–400)
Tumor location, *n* (%)	
Lower esophagus	30 (88.2)
Esophagogastric junction	4 (11.8)
Histology, *n* (%)	
Squamous cell carcinoma	29 (85.2)
Adenocarcinoma	4 (11.8)
Others	1 (2.9)
Pathological T stage, *n* (%)	
T1	2 (5.8)
T2	9 (26.5)
T3	23 (67.7)
Pathological N stage, *n* (%)	
N0	12 (35.3)
N1/2/3	14/5/3 (41.2/14.7/8.8)
Number of lymph nodes, median (range)	32 (15–78)
Length of thoracic drainage stay (days), median (range)	7 (4–25)
Length of postoperative hospital stay (days), median (range)	10 (7–28)

**Table 3:** Postoperative complications of the entire cohort

Complications description	Value
Anastomotic fistula, *n* (%)	1 (2.9)
Recurrent laryngeal nerve palsy, *n* (%)	2 (5.9)
Pneumonia, *n* (%)	3 (8.8)
Atrial fibrillation, *n* (%)	3 (8.8)
Chylothorax, *n* (%)	1 (2.9)
Delayed conduit emptying, *n* (%)	1 (2.9)

## Discussion

In this study, we describe our initial experiences and results in 34 consecutive patients with esophageal and gastroesophageal junction malignancies using an intrathoracic totally mechanical side‐to‐side: posterior‐to‐posterior linear and transverse stapled anastomosis. Minimally invasive esophagectomies seem to contribute significantly to a smooth postoperative recovery by reducing postoperative complications and improving quality of life.^3^, ^4^ Luketich *et al*. first described a MIE using a combined thoracoscopic esophageal mobilization followed by fashioning of the gastric conduit laparoscopically and construction of a cervical esophagogastric anastomosis.^21^ A randomized, controlled trial from Europe comparing MIE to open esophagectomy demonstrated the appropriateness of an oncologic resection utilizing MIE and highlighted significant improvements in short‐term outcomes over the open procedure.^12^ However, there has been a slow adaptation of the MIE procedure mainly due to the need to perform the dissection in different body cavities and the technical difficulty of the operation. This is especially relevant as to whether or not a cervical versus an intrathoracic anastomosis should be employed, as well as whether a hand sewn, circular stapled or linear stapled anastomosis is preferred.^12^, ^22^

A cervical anastomosis offers not only less severe morbidity associated with anastomotic complications but also technical simplicity when constructing the actual anastomosis. However, an intrathoracic anastomosis reduces the tension on the anastomosis for both the gastric conduit and the proximal esophagus, and is accompanied by a relatively well nourished conduit tissue, which subsequently would lead to a reduced incidence of anastomotic dehiscence. In addition, a cervical anastomosis may not guarantee an oncologically safe distal margin in some patients whose tumors have involved the gastroesophageal junction. In fact, some studies, including randomized controlled trials, suggest no significant difference in major surgical complications between the two types of anastomoses.^11^, ^23^ Nonetheless, many retrospective studies suggest that cervical anastomoses are associated with a higher risk of surgical complications, particularly anastomotic leakage and vocal cord paralysis.^24^, ^25^, ^26^ Our cumulative experience also confirms these findings, which led us to design this improved MIE‐IL procedure for lower esophageal and gastroesophageal junction cancers.

Methods for constructing the esophagogastric anastomosis in minimally invasive surgical procedures vary because of difficulties related to technically accessing the area. Multiple reports have compared anastomotic leak rates among various techniques. A recent meta‐analysis found lower leak rates with a linear stapled esophagogastric anastomosis compared to a completely hand‐sewn technique.^8^ A separate meta‐analysis by the same authors found no difference in leak rates between linear stapled and circular stapled esophagogastric anastomoses, but there were significant different stricture rates.^9^ A recent analysis using the Society of Thoracic Surgeons General Thoracic Database found an overall leak rate of 10.6% among 7595 esophagectomies, with rates of 12.3% and 9.3% for cervical and intrathoracic anastomosis, respectively.^25^ According to these data, linear stapled side‐to‐side anastomosis is one of the most reasonable intrathoracic anastomosis procedures to perform during a minimally invasive Ivor Lewis esophagectomy. However, there is a certain technical skill required to close the remaining defect after stapling the linear anastomosis, which increases the technical difficulty of the operation. Due to this difficulty, a side‐to‐side liner stapled anastomosis is mainly used for McKeown or open Ivor Lewis procedure.^27^ In addition, the retention of a gastric conduit stump increases the incidence of an ischemic anastomotic fistula forming. The advantages mentioned above with an intrathoracic side‐to‐side anastomosis may therefore outweigh other options. Therefore, in an attempt to reduce the difficulty of this surgical procedure, we utilized an improved TM‐STS technique.

An analysis of the results from our current practice demonstrates outstandingly low rates of anastomotic leakage when we compared to other high‐volume, minimally invasive centers. We have identified only one anastomotic leak in 34 patients undergoing a MIE‐IL utilizing a linear stapler with a 60‐mm, side‐to‐side anastomosis. There are some possible reasons for our low leak rates associated with this improved technique. First, the technique utilizes a relatively well‐vascularized area on the posterior side of the esophagus and stomach where the gastric conduit staple line resides instead of at the end of the esophagus. Second, a 3 cm proximal gastric conduit was excised intraoperatively and the fundus of the stomach was formed at that time, which may reduce the influence of gastric juice reflux on the anastomosis and ensure sufficient blood flow to the anastomosis. Third, the large anastomotic opening spreads the distribution of pressure when the stapler is applied and reduces the shear forces on the anastomosis that tend to pull side to side.

Petrin *et al*. reported that the incidence of anastomotic stenosis was inversely proportional to the diameter of the annular stapler, and the incidence of stenosis was 12.3% in 187 patients, among whom no anastomotic stenosis occurred in patients with an anastomotic circumference greater than 29 mm.^28^ Gupta and colleagues removed a 3 cm long and 2 cm wide crescent of anterior gastric wall tissue with a predesigned hand‐sewn anastomosis, and speculated that a wide cross‐sectional diameter of the anastomosis might reduce the incidence of anastomotic stenosis. Finally, their new anastomotic procedure reduced the incidence of anastomotic stenosis from 29% in the conventional control group to 9% in the treatment group (*P* = 0.02).^29^ Subsequent studies have shown a stricture rate ranging from 13% to 20% with no report on stricture rates in other larger series.^30^ Our low stricture rate can largely be attributed to our side‐to‐side stapled anastomotic technique, which ensures a large enough anastomotic area fundamentally, thus reducing the incidence of anastomotic stenosis.

The results of this improved TM‐STS technique reveal that it can be employed safely and effectively in patients with lower esophageal as well as Siewert types I/II gastroesophageal junction carcinoma during MIE‐IL. The procedure described in this study utilized a linear 60 mm stapled intrathoracic anastomosis and was associated with excellent outcomes, including low leak and stricture rates. However, the optimal approach and anastomotic type may not be determined unless evaluated by a randomized trial comparing these techniques.

In our study, there were several limitations that should be mentioned when interpreting our results. Whereas the leak rate is low utilizing this technique for a minimally invasive Ivor Lewis esophagectomy, it is a technically demanding operation and requires more minimally invasive skills than a cervical anastomosis. Although a relatively simple technique, nevertheless a learning curve may be required. Of note, in our series, reoperation for an anastomotic leak was not needed. In addition, there was no patients requiring dilatation for anastomotic stricture within the short‐term follow‐up period; however, long‐term follow‐up outcomes are unknown.

In conclusion, this improved TM‐STS procedure is relatively adaptable and reproducible with promising short‐term outcomes, which can be considered as one of the alternative procedure for patients with lower esophageal as well as Siewert types I/II gastroesophageal junction carcinoma. The long‐term outcomes need to be investigated in the future, including a study of the effect of our technique on a patient's esophageal‐specific quality of life.
